# *PeHVA22* gene family in passion fruit (*Passiflora edulis*): initial characterization and expression profiling diversity

**DOI:** 10.3389/fpls.2023.1279001

**Published:** 2024-01-19

**Authors:** Zhimin Hou, Jianxiang Liang, Xinkai Cai, Jingting Lin, Xiaomei Wang, Ruoyu Liu, Lin Lu, Gaifeng Chai, Chang An, Shengzhen Chen, Yuan Qin, Ping Zheng

**Affiliations:** ^1^College of Life Sciences, Fujian Provincial Key Laboratory of Haixia Applied Plant Systems Biology, Center for Genomics and Biotechnology, Fujian Agriculture and Forestry University, Fuzhou, China; ^2^Center for Viticulture and Enology, School of Agriculture and Biology, Shanghai Jiao Tong University, Shanghai, China; ^3^Horticulture Research Institute, Guangxi Academy of Agricultural Sciences, Nanning Investigation Station of South Subtropical Fruit Trees, Ministry of Agriculture, Nanning, China; ^4^Pingtan Science and Technology Research Institute, College of Marine Sciences, Fujian Agriculture and Forestry University, Fuzhou, China

**Keywords:** passion fruit, *HVA22* genes, phytohormone and stress response, floral development, expression analysis

## Abstract

Passion fruit, an economically valuable fruit crop, is highly vulnerable to adverse climate conditions. The *HVA22* genes, recognized as abscisic acid (ABA) and stress-inducible, play vital roles in stress response and growth regulation in diverse eukaryotic organisms. Here, six *HVA22* genes were firstly identified in passion fruit genome and all predicted to be localized within the endoplasmic reticulum. Phylogenetic analyses showed that all PeHVA22s were divided into four subgroups. The gene structural features of *PeHVA22* genes clustered in the same subgroup were relatively conserved, while the gene structure characteristics of *PeHVA22s* from different subgroups varied significantly. *PeHVA22A* and *PeHVA22C* closely clustered with barley *HVA22* in Group II, were also induced by ABA and drought stress treatment, suggesting conserved roles similar to barley *HVA22*. Meanwhile, most *PeHVA22s* exhibited induced expression post-drought treatment but were suppressed under salt, low and high-temperature conditions, indicating a unique role in drought response. Additionally, *PeHVA22s* displayed tissue-specific expression patterns across diverse tissues, except for *PeHVA22B* which maybe a pseudogene. Notably, *PeHVA22C*, *PeHVA22E*, and *PeHVA22F* predominantly expressed in fruit, indicating their involvement in fruit development. Almost all *PeHVA22s* showed variable expression at different developmental stages of stamens or ovules, implying their roles in passion fruit’s sexual reproduction. The intricate roles of *PeHVA22s* may result from diverse regulatory factors including transcription factors and CREs related to plant growth and development, hormone and stress responsiveness. These observations highlighted that *PeHVA22s* might play conserved roles in ABA response and drought stress tolerance, and also be participated in the regulation of passion fruit growth and floral development.

## 1 Introduction

Passion fruit (*Passiflora edulis*), belonging to the Passifloraceae family, is one of the popular tropical fruit crops. Because of its great edible, medicinal and ornamental value, passion fruit has become one of the most important tropical edible flavorful fruits (Lopez-Vargas et al., 2013). The flower of passion fruit is large and peculiar, making it also an ideal garden ornamental plant (Faleiro et al., 2019). Passion fruits are mainly adapted to climates ranging from cool subtropical to warm tropical but exhibit high sensitivity to diverse stressful conditions such as water deficiency, low temperatures, and high temperatures (Chebet et al., 2023). Therefore, investigating genes involved in stress response regulation holds significant importance for developing molecular breeding strategies in passion fruit.

Abscisic acid (ABA) plays a pivotal role in plant stress responses by regulating an extensive array of stress-responsive genes, enabling plants to effectively manage dynamically changing climates (Chandler, 1994). *HVA22*, a distinctive ABA-induced gene, was initially isolated from the aleurone layer of barley (*Hordeum vulgare*) and identified as a stress-induced gene (Shen et al., 1993; Shen et al., 1996; Shen et al., 2001). To date, over 355 homologs of *HVA22* have been described in eukaryotic organisms, including plants, animals, protozoa, and fungi, with the exception of prokaryotes (Shen et al., 2001; Guo and Ho, 2008; Sharon and Suvarna, 2017). Across all homologs, the TB2/DP1/HVA22 domain regions within the HVA22 protein structure exhibit a high degree of conservation (Guo and Ho, 2008; Sharon and Suvarna, 2017). The regulation of *HVA22* expression in plants has been comprehensively elucidated in response to both biotic and abiotic stress inductions. The promoter of the barley *HVA22* gene contains an ABA response complex, serving as a requisite and adequate element for mediating ABA-induced gene induction (Shen and Ho, 1995). Exposure to ABA, cold, salt, and drought stresses induces the transcript of *HVA22* in barley leaves (Shen et al., 1993; Shen et al., 2001). Likewise, the promoter of *HVA22* in rice is stress-inducible and controls drought tolerance by regulating the expression of osmotic related genes (Xiao et al., 2009). In *Arabidopsis*, the identification of five *HVA22* homologs revealed distinctive expression levels across diverse tissues when subjected to ABA and various abiotic stress treatments (Chen et al., 2002). Moreover, ABA-induced *HVA22* is also associated with seed dormancy, seed germination, and seedling growth in barley (Shen et al., 2001; Brands and Ho, 2002; Guo and Ho, 2008). The RNAi mutant of *AtHVA22d* exhibits enhanced autophagy capability, resulting in smaller siliques and reduced yield (Shen and Ho, 1995). It is conceivable that plant *HVA22* also participates in plant growth and development.

Taking advance to the published passion fruit genome (Ma et al., 2021; Xia et al., 2021), the complexity of diverse gene families has been systematically analyzed (Liang et al., 2022; Liang et al., 2023). Investigations into the sequence divergence and unique expression patterns within the stress-induced *HVA22* gene family are positioned to enhance our understanding of their functional roles. In this study, bioinformatic methods were employed to identify the *HVA22* gene family from the whole-genome and to elucidate the phylogenetic evolution of passion fruit. We identified six *HVA22* genes in the passion fruit genome. The gene structure, conserved motifs, phylogenetic relationships, *Cis*-regulatory elements (CREs), and expression profiling across diverse tissues were investigated. Meanwhile, the expression pattern of *PeHVA22s* under various phytohormone and stress treatments were also analyzed. Overall, this investigation will facilitate subsequent functional examinations of *PeHVA22s* and provide clues for improvement of the passion fruit breeding.

## 2 Materials and methods

### 2.1 Plant materials, growth conditions and stress treatments

The plant materials were obtained from the passion fruit breeding group at Fujian Agriculture and Forestry University, focusing on the “Tai Nong” variety. Seeds were germinated in a greenhouse under controlled conditions (temperature: 30 ± 1^°^C, photoperiod: 16-h light/8-h dark) with a relative humidity of 70%. The seedings with two true leaves grown in the growth chamber for two months.

Two-month-old passion fruit plants were subjected to various treatments, including phytohormone (ABA and GA) and abiotic stresses (drought, salt, cold, and heat). As to phytohormone treatments, healthy plants were exposed to ABA and GA at concentrations of 100 μM. Drought and salt stress were induced using mannitol (200 mM) and Nacl (200 mM) for healthy seedings. For cold stress (8°C) and heat stress (45°C) treatment, healthy plants were placed in an incubator with a 16/8 h day/night cycle. Leaf samples were collected at 0h, 12h, 24h, and 48h during stress treatments from three independent seedings each. Subsequently, all obtained samples were promptly stored in liquid nitrogen before total RNA extraction. Additionally, untreated plants were used as controls.

### 2.2 RNA extraction and qRT–PCR analysis

The RNA extraction was performed following manufacturer’s guidance utilizing plant total RNA extraction Kits (Omega Bio-Tek, Shanghai, China). RNA quality was assessed using both a spectrophotometer (NanoDrop, United States) and agarose gel electrophoresis. Reverse transcription into cDNA was performed following the protocol of the reverse transcription kit (TaKaRa, Beijing, China). qRT-PCR assays were conducted using the SYBR Premix Ex Taq II (TaKaRa, Beijing, China). The qRT-PCR program included initial denaturation at 95°C for 30 seconds; followed by 40 cycles of denaturation at 95°C for 5 seconds and annealing and extension at 60°C for 34 seconds; finally, denaturation at 95°C for 15 seconds. Each sample underwent three replicates. The relative expression levels of *PeHVA22* genes were determined using the reference gene EF1a (Zhao et al., 2022). After designing primers for all *PeHVA22* genes using the PrimerQuest tool, we utilized the BLAST tool to align the primer sequences to the passion fruit genome coding sequence (CDS). Based on the alignment results, we examined whether the primers designed for each gene were specific (**Supplementary Table 6**).

### 2.3 Identification and characterization analysis of *HVA22* genes in passion fruit

The passion fruit genome and protein sequences were retrieved from the National Genomics Data Center (NGDC) under the accession number GWHAZTM00000000. The protein sequence of the Arabidopsis *HVA22* family genes and barley *HVA22* gene were downloaded from the NCBI database (https://www.ncbi.nlm.nih.gov) and used as initial queries for BLASTP searches. Meanwhile, the hidden Markov model (HMM) of the TB2/DP1/HVA22 domain (PF03134) was acquired from the PFAM database (http://pfam.xfam.org/) and served as the initial model for the HMMER (v3.3) search (http://hmmer.janelia.org/) within the local passion fruit protein database, with an E-value threshold of 10^-5^. After filtering out the redundant sequences, all the candidate genes were further confirmed by the NCBI Conserved Domain Database (https://www.ncbi.nlm.nih.gov/cdd/) (E-value < 1 × 10^−5^) and the SMART (http://smart.embl-heidelberg.de/) database (Letunic et al., 2012). Genes lacking the TB2/DP structural domain were excluded from the analysis. The ExPASy Server (https://web.expasy.org/protparam/) (Wilkins et al., 1999) used to calculate the protein physicochemical parameters, such as molecular weight (MW), isoelectric point (PI), and grand average of hydropathicity (GRAVY). Meanwhile, subcellular localization of PeHVA22 proteins were predicted with Cell-PLoc 2.0 (http://www.csbio.sjtu.edu.cn/bioinf/Cell-PLoc-2/) (Chou and Shen, 2010).

### 2.4 Multiple sequence alignment and phylogenetic analysis

MUSCLE software was employed to compare the multiple protein sequences of the HVA22 genes in passion fruit and Arabidopsis (Edgar, 2004). Phylogenetic tree analysis of related proteins (*Passiflora edulis*, *Arabidopsis thaliana*, *Solanum lycopersicum*, *Solanum tuberosum*, *Oryza sativa*, *Hordeum vulgare* and *Zea mays*) was performed using MEGA 11 software with the maximum likelihood method (Tamura et al., 2021). The Evolview online website (http://evolgenius.info/#) was used to visualize the evolutionary relationship tree.

### 2.5 Three-dimensional structural modeling of PeHVA22 family proteins

Protein models homologous to passion fruit HVA22 protein were searched using the PDB database (http://www.rcsb.org/). The protein tertiary structure was predicted by homology modeling, using the SWISS-MODEL with default parameters (https://www.swissmodel.expasy.org/), and the ConSurf (https://consurf.tau.ac.il/) was used to analyze the conservative structure (Ashkenazy et al., 2016). The 3D protein model structure was visualized and manipulated by PyMOL (Delano, 2002). Finally, we use Protter (http://wlab.ethz.ch/protter/start/) to visualize the topological structure of the protein (Omasits et al., 2014).

### 2.6 Gene structure, conserved motif and CREs prediction analysis

The intron-exon distributions of the passion fruit *HVA22* genes were obtained using GFF annotation files from the passion fruit genome. Protein sequence analysis was performed using the MEME online program (http://meme-suite.org/) (Bailey et al., 2009) to identify the conserved motifs in PeHVA22 proteins. The parameters were set as follows: znr (the occurrences of each functional domain in each sequence was variable) as the distribution type of the structure domain in the sequence, and 10 motifs were calculated. The upstream 2,000 bp sequence of each *PeHVA22* gene was retrieved using TBtools software (Chen et al., 2020) based on the genomic full-length DNA sequences of *PeHVA22s*. Subsequently, the PlantCare database (http://bioinformatics.psb.ugent.be/webtools/plantcare/html/) (Lescot et al., 2002) was employed to predict cis-regulatory elements (CREs) in the putative promoter region of *PeHVA22s*. The results were visualized using TBtools software (Chen et al., 2020).

### 2.7 Prediction of transcription factor networks

The prediction of the transcription factor network was conducted following the approach outlined by Wang et al. (Wang et al., 2019) with minor modifications. The 2,000 bp upstream sequence from each *PeHVA22* gene was extracted and submitted to the Plant Transcriptional Regulatory Map (PTRM) website (http://plantregmap.cbi.pku.edu.cn/regulation_prediction.php) to predict potential transcription factors involved in the regulation of the *PeHVA22* genes, with the setting of p-value ≤ 1e^-6^. The Cytoscape 3.6 software was employed to visualize the transcription factor regulatory network (Kohl et al., 2011).

## 3 Results

### 3.1 Identification and characterization of *HVA22* genes in passion fruit

To identify *HVA22* genes in the passion fruit whole genome, we initially conducted BLAST and Hidden Markov Model searches. Subsequently, SMART and NCBI-CDD online tools were employed to validate the TB2/DP1/HVA22 conserved domains of these candidate proteins. After removing the redundant sequences, a total of six *PeHVA22* genes were identified in passion fruit, designated as *PeHVA22A* to *PeHVA22F* according to their chromosomal distribution. *PeHVA22A* - *PeHVA22D* were located on LG01, while *PeHVA22E* and *PeHVA22F* were on LG04 and LG06, respectively (**Table 1**). Physicochemical property analyses revealed that the amino acid length of six PeHVA22 proteins ranged from 139 aa (PeHVA22C) to 285 aa (PeHVA22D), with the corresponding molecular weight varying from 16,233.86 to 31,870.92 Da. The predicted proteins’ isoelectric points (pI) ranged from 7.01 (PeHVA22E) to 9.32 (PeHVA22F). Expect for PeHVA22C and PeHVA22F, the grand average of hydropathicity score (GRAVY) for the other proteins were negative, indicating that they were dominantly hydrophilic. The aliphatic amino acid index (A.I.) of PeHVA22 proteins ranged from 63.12 (PeHVA22D) and 109.94 (PeHVA22F) Stability index calculations predicted that half of these proteins including PeHVA22A, PeHVA22C and PeHVA22F were stable, while the other half was unstable. Subcellular localization predictions indicated that all PeHVA22 proteins were localized in endoplasmic reticulum.

**Table 1:** Molecular characteristics of *PeHVA22* genes in Passion Fruit.

Gene Name	Gene ID	Chromosome	Size (aa)	MW (Da)	pI	GRAVY	A.I.	Stability	Predicted Location	Quantity of exons
*PeHVA22A*	*P_edulia010000431.g*	LG01	142	16596.36	9.1	-0.015	89.86	S	Endoplasmic reticulum	5
*PeHVA22B*	*P_edulia010001434.g*	LG01	251	28973.06	8.23	-0.355	85.5	U	Endoplasmic reticulum	7
*PeHVA22C*	*P_edulia010002149.g*	LG01	139	16233.86	7.99	0.036	97.48	S	Endoplasmic reticulum	6
*PeHVA22D*	*P_edulia010004731.g*	LG01	285	31870.92	8.77	-0.552	63.12	U	Endoplasmic reticulum	7
*PeHVA22E*	*P_edulia040010224.g*	LG04	193	22096.54	7.01	-0.032	92.02	U	Endoplasmic reticulum	10
*PeHVA22F*	*P_edulia060015668.g*	LG06	156	17969.35	9.32	0.308	109.94	S	Endoplasmic reticulum	5

MW, molecular weight; pI, isoelectric point; GRAVY, grand average of hydropathicity score; A.I, aliphatic index.

### 3.2 Multiple sequence alignment and phylogenetic analysis of PeHVA22 proteins

**Figure 1:**
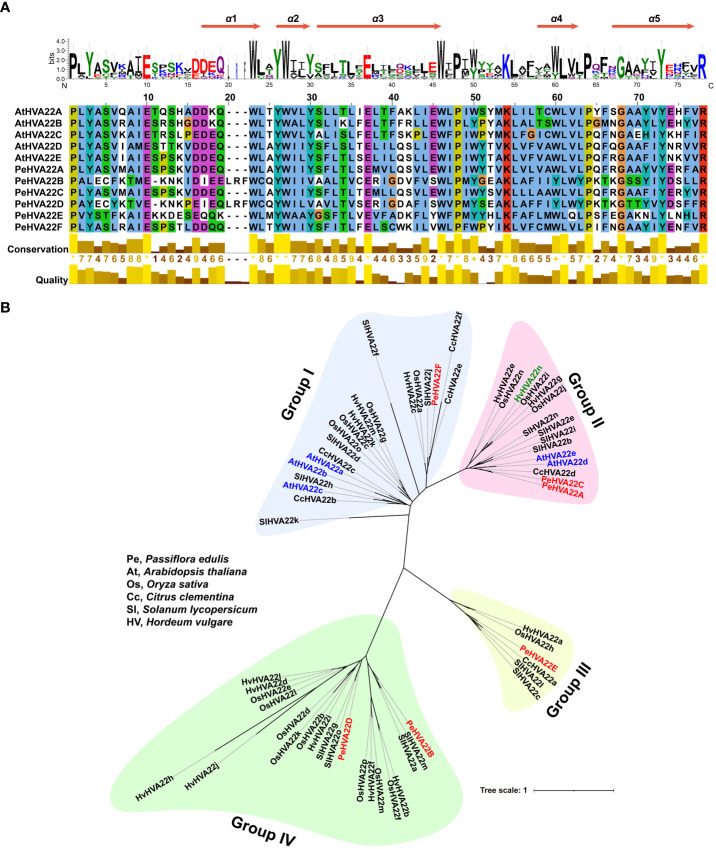
**(A)** Multiple sequence alignment of TB2/DP1/HVA22 domains of PeHVA22 and AtHVA22 proteins. Five α-helix bundles in the conserved TB2/DP1/HVA22 domain regions were identified in all proteins. **(B)** Unrooted maximum likelihood (ML) phylogenetic tree of all HVA22 proteins from Pe (*Passiflora edulis*), At (*Arabidopsis thaliana*), Sl (*Solanum lycopersicum*), Os (*Oryza sativa*), Hv (*Hordeum vulgare*, barley) and Cc (*Citrus clementina*). The phylogenetic tree was constructed by RAxML software with a setting of 1000 bootstrap replicates. PeHVA22s, AtHVA22s, and the reported “barley HVA22” gene (Shen et al., 2001) renamed as “HvHVA22n” were highlighted with red, blue and light green color, respectively.

Multiple sequence alignments were conducted on six PeHVA22s and five Arabidopsis AtHVA22 proteins. The TB2/DP1/HVA22 conserved domain and five α-helix bundles within the conserved domain regions were identified in all these homologous proteins, underscoring the conservation of HVA22 in dicotyledons (**Figure 1A**). To assess evolutionary relationships, a maximum likelihood (ML) phylogenetic tree was constructed using 62 HVA22 protein sequences from passion fruit (6 PeHVA22s), *Arabidopsis* (5 AtHVA22s) (Chen et al., 2002), *Solanum lycopersicum* (15 SlHVA22s) (Wai et al., 2022), *Oryza sativa* (16 OsHVA22s) (Wai et al., 2022), *Citrus clementina* (6 CcHVA22s) (Gomes Ferreira et al., 2019) and barley (14 HvHVA22s) (Shen et al., 2001) (**Supplementary Table 1**). The phylogenetic analysis classified all HVA22 proteins into four subgroups, named as Group I to Group IV (**Figure 1B**). Group IV had the highest number of members with 21 genes, followed by Group I (20) and Group II (15). Group III was the smallest subgroup, consisting of six members. AtHVA22s and CcHVA22s were predominantly found in Group I and Group II, while the HVA22 proteins of other species were unevenly distributed across all four subgroups. Among that, PeHVA22A and PeHVA22C were closely clustered with AtHVA22d and AtHVA22e in the same subgroup, which also included barley HVA22 (HvHVA22n). Additionally, PeHVA22B and PeHVA22D were clustered together in Group IV with rice OsHVA22m (OsHLP1, HVA22-like protein 1) (Meng et al., 2022). Homologous genes exist in the same subclade might perform similar functions (Liu et al., 2018).

### 3.3 Comparative tertiary structure modelling of PeHVA22 proteins

**Figure 2:**
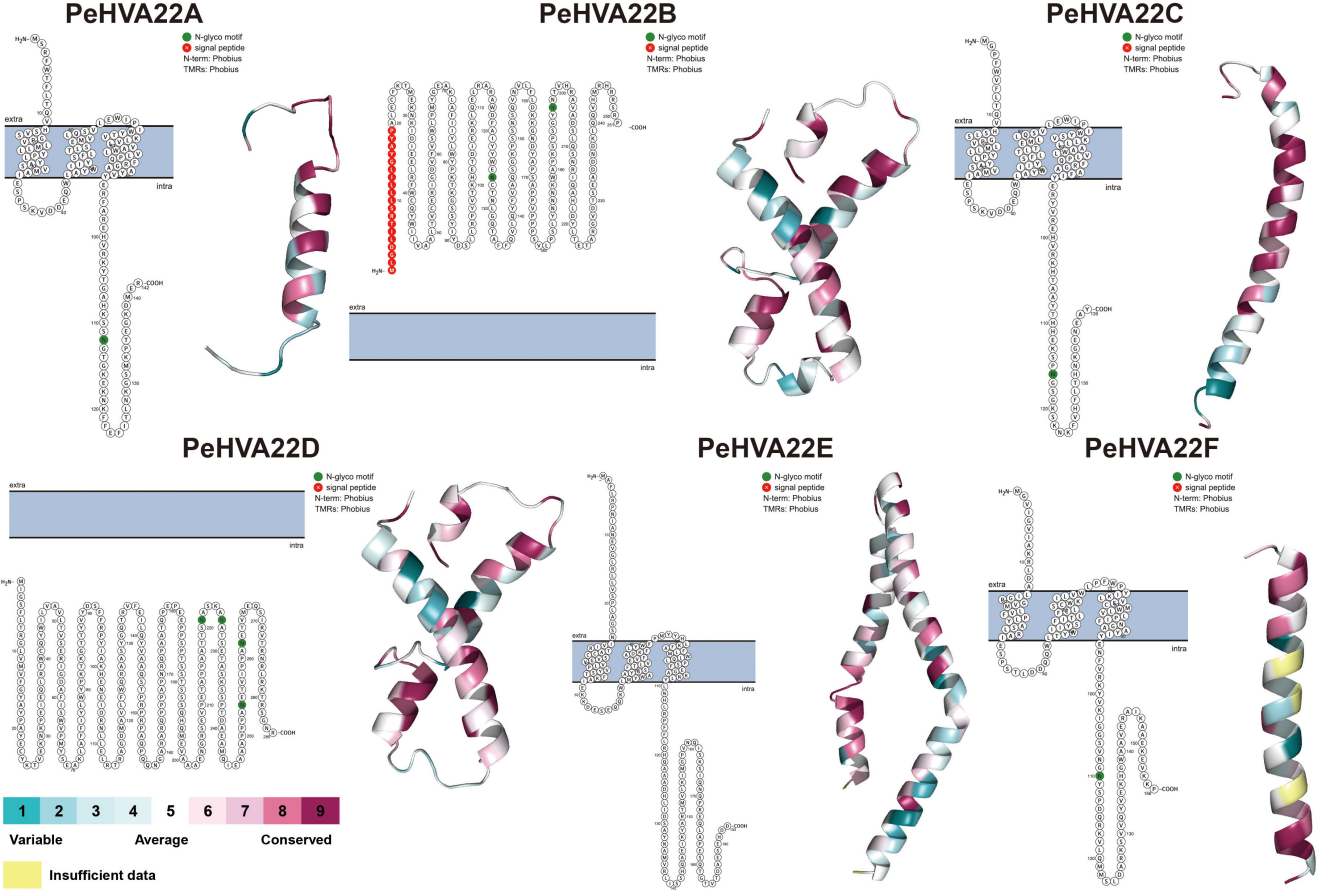
Predicted protein topology map and 3D structures of PeHVA22 protein sequences. The blue bars represented the plasma membrane, and the extra- and the intra-cellular regions were specified.

The generation of protein topology map and 3D structure is essential for gaining a comprehensive understanding of protein localization, structure, and function. Initially, the membrane topology model for PeHVA22 proteins was predicted using Protter software (Omasits et al., 2014). With the exception of PeHVA22B and PeHVA22D proteins, located entirely outside and inside the membrane respectively, the remaining PeHVA22 proteins exhibited three membrane crossings each (**Figure 2**). Notably, all PeHVA22 proteins, except PeHVA22E, possessed 1-2 N-glycosylation motifs. Homology modeling of PeHVA22 proteins was conducted using the SWISS-MODEL database, resulting in the prediction of 3D structural models of six PeHVA22 proteins (**Figure 2**). Each PeHVA22 protein comprised α-helix and β-sheet structures, with variations in their overall architectures, suggesting potential differences in biological functions. Taken together, the homology models of these proteins provided a preliminary basis for further understanding the molecular functions of PeHVA22 proteins.

### 3.4 Gene structure, conserved motif and CREs analysis of *PeHVA22* genes

**Figure 3:**
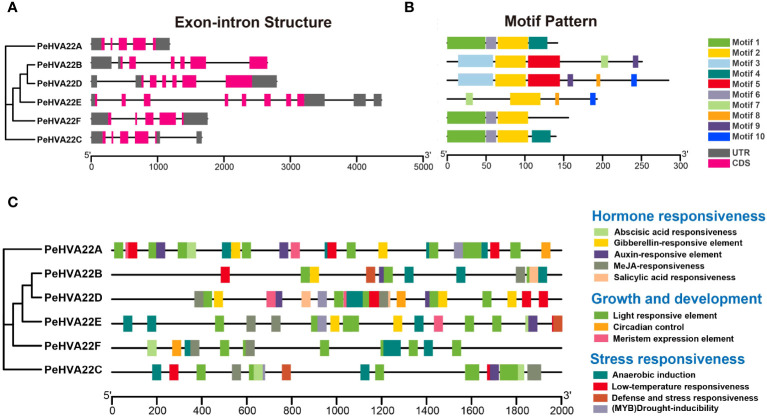
**(A)** Gene structures of *PeHVA22s*. Black lines represented introns, rose red rectangles represented exons, and grey rectangles represented untranslated regions (UTRs). The scale bar indicated 1000 bp; **(B)** Distribution of conserved motifs in PeHVA22 proteins. Different motifs are displayed by boxes with different colors. **(C)** Distribution of CREs identified in the 2000 bp upstream putative promoter region of *PeHVA22s*.

The exon-intron structure and conserved motif compositions were analyzed to further explore the evolution of the *PeHVA22* gene family. Genomic structural analysis of exon-intron organizations in the *PeHVA22s* revealed a variation in the number of exons, ranging from five to eight (**Figure 3A**). *PeHVA22E* exhibited the highest number of exons (eight), followed by *PeHVA22B* and *PeHVA22D* (six exons), while *PeHVA22A*, *PeHVA22C*, and *PeHVA22F* contained five exons. To explore the diversity of *PeHVA22s*, the putative motifs were predicted and identified by MEME, with a setting of 10 motifs (Bailey et al., 2009). The number of motifs in PeHVA22 members ranged from three to six (**Figure 3B**), and all PeHVA22 members contained Motif 2, suggesting that the function of Motif 2 was conserved. Unique motifs were also identified for specific PeHVA22 members, such as Motif 1 and Motif 6 for PeHVA22A, PeHVA22C, and PeHVA22F, Motif 3 and Motif 5 for PeHVA22B and PeHVA22D, and Motif 8 and Motif 10 for PeHVA22D and PeHVA22E. Variations in motifs and gene structures among PeHVA22s might contribute to functional diversity, while PeHVA22s with similar features may share common functions.

*Cis*-regulatory elements (CREs) are non-coding DNA sequences located in gene promoter regions, regulating the transcription of associated genes (Zhao et al., 2018). To explore the potential biological functions of *PeHVA22* genes, we extracted the 2000 bp regions upstream of all *PeHVA22* genes transcription start site and detected their CREs using the PlantCARE program (Lescot et al., 2002). The identified CREs in the promoter regions of *PeHVA22* genes were mainly divided into three categories: hormone responsiveness elements, growth and development elements, and stress responsiveness elements (**Figure 3C**, **Supplementary Tables 2**, **3**). In the hormone responsiveness category, the core CREs were associated with the regulations of abscisic acid (ABRE), gibberellin (GARE-motif, TATC-box, and P-box), auxin (TGA-element), methyl jasmonate (CGTCA-motif and TGACG-motif) and salicylic acid (TCA-element). These results indicated that the expression of *PeHVA22s* were subjected to the regulation of diverse phytohormones. Notably, abscisic acid responsiveness-related CREs were present in the promoter regions of almost all *PeHVA22s*, except for *PeHVA22B*, but were particularly abundant in *PeHVA22A* and *PeHVA22C* (**Supplementary Table 2**). Besides, gibberellin responsiveness-associated CREs were found in the promoter regions of *PeHVA22A*, *PeHVA22B*, *PeHVA22D* and *PeHVA22E*. Some stress-responsive elements were also predicted, including anaerobic inducible element (ARE), low temperature response element (LTR), (MYB) drought-inducibility (MBS), defense and stress response elements (TC-rich repeats), and wound-responsive element (WUN-motif). Additionally, various growth and development response elements were also identified, such as diverse light-responsive elements (G-box, AE-box, TCT-motif, GATA-motif, and LAMP-element), circadian control (MSA-like and circadian), and meristem expression elements (CAT-box). These findings suggested that *PeHVA22s* play a role in the growth and development regulation of passion fruit and response to diverse environmental stresses.

### 3.5 Transcription factor regulatory network of *PeHVA22* genes

**Figure 4:**
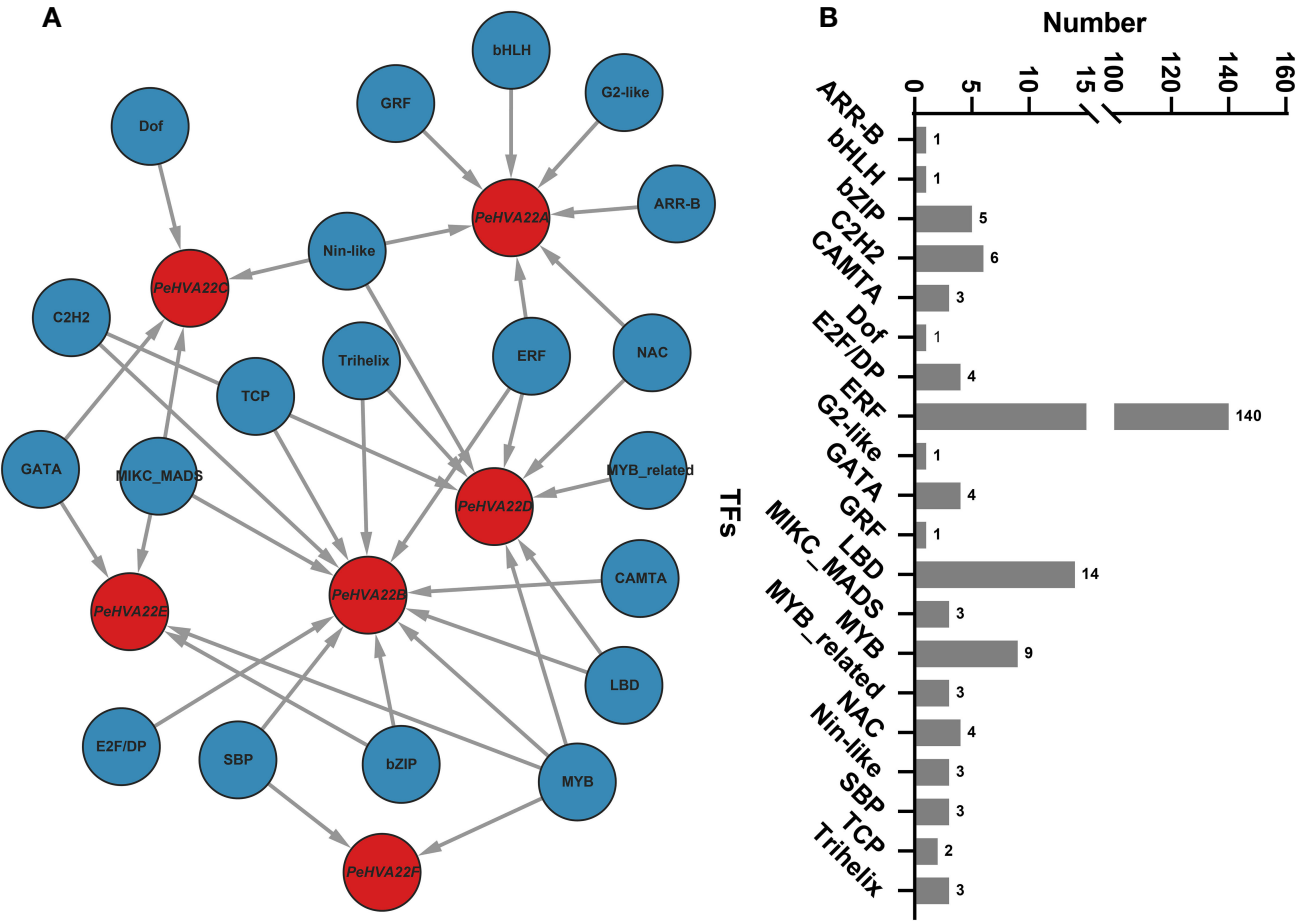
The putative transcription factor regulatory network of *PeHVA22* genes. **(A)** The transcriptional regulatory network of *PeHVA22* genes predicted by PTRM tool and visualized with the Cytoscape 3.6 software. **(B)** The statistic diagram of the predicted transcription factors.

To explore the potential TF regulatory network of the *PeHVA22* genes, the putative promoter sequences of the six *PeHVA22* genes were extracted and used for prediction analysis at the PTRM website (http://plantregmap.gao-lab.org/) (Tian et al., 2020). The results revealed that diverse TF families, primarily including *ERF*, *LBD*, *MYB*, *C2H2* and *bZIP*, were involved in the regulating of *PeHVA22* genes (**Figure 4**). Among these TFs, the *ERF* members were the most abundant (140), followed by *LBD* (14) and *MYB* members (9) (**Figure 4B**). Among all the *PeHVA22* genes, *PeHVA22D* was the one targeted by most TFs (132), followed by *PeHVA22A* (32), while *PeHVA22B* was the one targeted by most classes of TFs (11) and followed by *PeHVA22D* (8) (**Supplementary Table 4**). In addition, many TFs involved in stress response were also identified such as *NAC, Dof* and *bZIP* TFs. Meanwhile, numerous TFs related with plant growth and development were also identified including the *LBD*, *MIKC_MADS*, and *SBP* TFs (**Figure 4A**).

### 3.6 Expression profile of *PeHVA22* genes in response to phytohormone and abiotic stresses treatments

**Figure 5:**
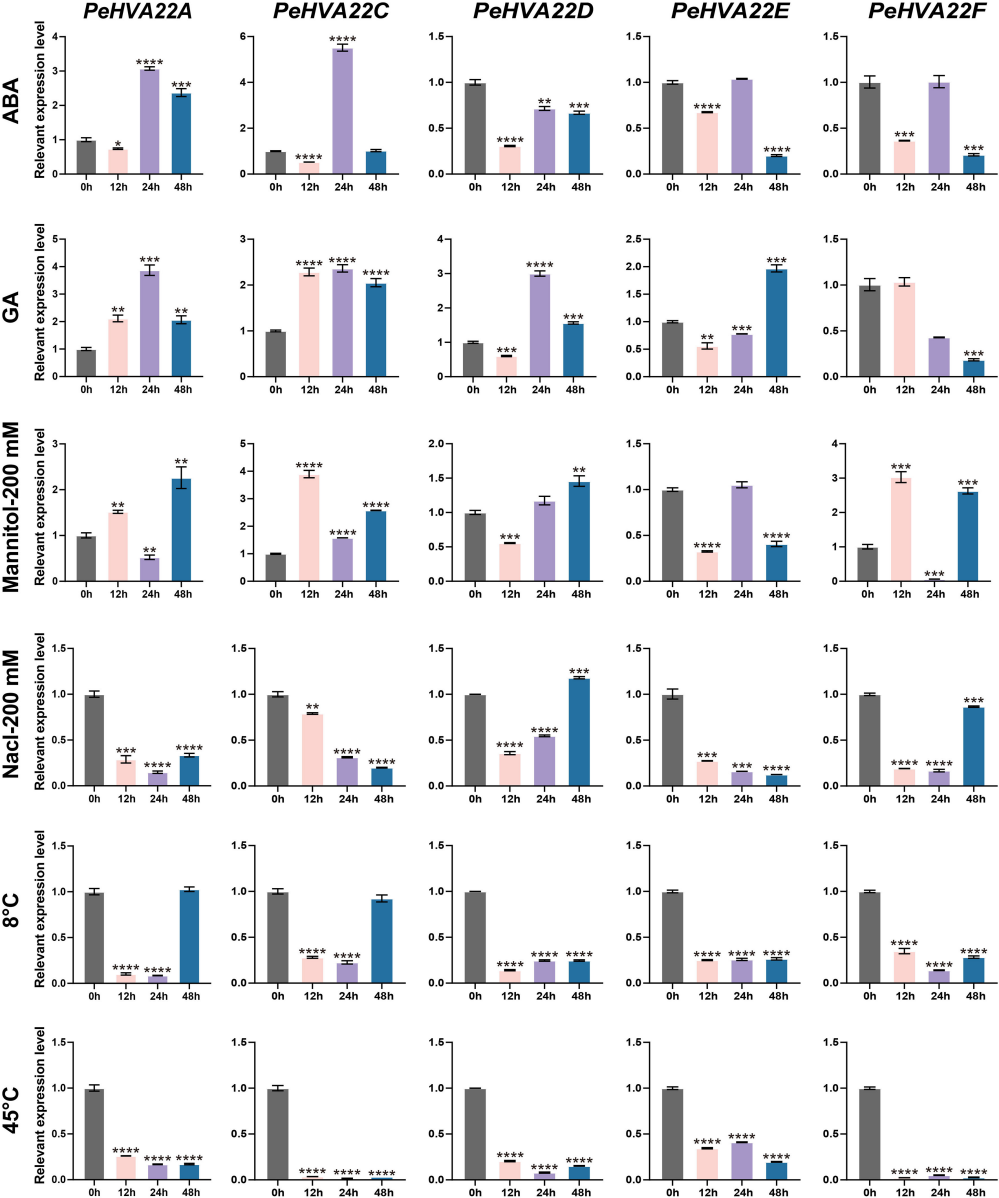
Expression patterns of *PeHVA22* genes in response to phytohormones and stress treatments. Each sample was collected at 0, 12, 24 and 48h after treatments. Significant differences were analyzed by the Student’s t-test (*p-value < 0.05, **p-value < 0.01, ***p-value < 0.001, and **** p-value < 0.0001).

To elucidate the response patterns of *PeHVA22s* to phytohormone and abiotic stresses, qRT-PCR was conducted to determine the relative expression profiles of *PeHVA22* genes in leaves of plant treated with different phytohormones (ABA and GA) and abiotic stresses (drought, salt cold, and heat) at different time points. All *PeHVA22* genes, except for *PeHVA22B*, which was not detectable, exhibited distinct response patterns under these treatments (**Figure 5**). In the ABA treatment, the expression of *PeHVA22A* and *PeHVA22C* was slightly repressed at 12 h after treatment and then significantly enhanced or induced at 24 h after treatment, while that of *PeHVA22D*, *PeHVA22E*, *PeHVA22F* was down-regulated at different time points. Upon GA treatment, the expression of *PeHVA22A* and *PeHVA22C* were enhanced from 12 h after treatment, while the expression level of *PeHVA22D* and *PeHVA22E* were initially decreased and subsequently increased, and the expression of *PeHVA22F* was repressed with a descending trend. In the drought (200 mM Mannitol) stress treatment, *PeHVA22A*, *PeHVA22C*, and *PeHVA22F* displayed similar expression profiles, initially increasing, then decreasing, and then increasing. The relative expression level of *PeHVA22D* was initially down-regulated and subsequently up-regulated. The relative expression of *PeHVA22E* was decreased at 12h and 48h after treatment, while increased at 24h after treatment. Under the salt (200 mM Nacl) treatment, the relative expression level of *PeHVA22A*, *PeHVA22C*, and *PeHVA22E* were down-regulated with treatment time. *PeHVA22D* and *PeHVA22F* showed similar expression patterns, which was initially decreased and then increased. In the cold (8°C) treatment, the expression of *PeHVA22A* and *PeHVA22C* were suppressed at 12 h and 24 h of treatment and subsequently restored to the initial level. The relative expression level of *PeHVA22D*, *PeHVA22E*, and *PeHVA22F* were down-regulated throughout the treatment. Additionally, all five *PeHVA22* genes showed a descending response pattern under heat (45°C) treatment. These results suggested that *PeHVA22* genes might function in response to phytohormones and abiotic stresses in different ways.

### 3.7 Expression patterns of *PeHVA22* genes in six representative passion fruit tissues

**Figure 6:**
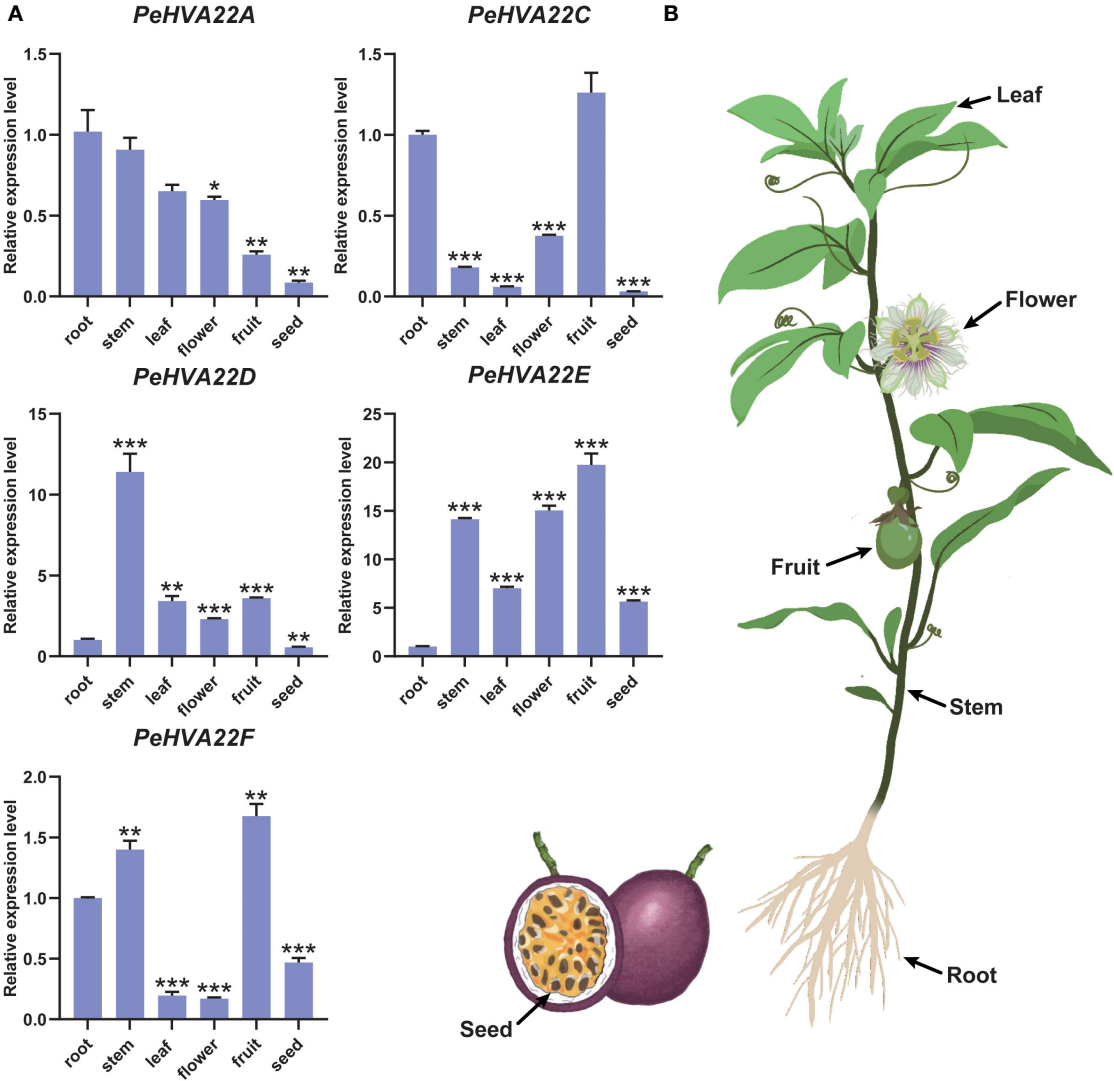
Tissue-specific expression profiles of *PeHVA22s* in passion fruit. **(A)** qRT-PCR analysis of the *PeHVA22* genes in six representative tissues including roots, stems, leaves, flowers, fruits and seeds. Significant differences were analyzed by the Student’s t-test (**p-value < 0.01, ***p-value < 0.001, and **** p-value < 0.0001). **(B)** Schematic illustration of the passion fruit plant showing collected tissues.

To explore the tissue-specific expression patterns of *PeHVA22* genes in passion fruit, we analyzed the expression profiles of each *PeHVA22* gene in six representative tissues (roots, stems, leaves, flowers, fruits, and seeds) using qRT-PCR. Expression levels were normalized relative to roots (control) in various tissues. The results revealed distinct tissue-specific expression patterns for *PeHVA22* genes (**Figure 6**). *PeHVA22A* exhibited preferentially higher expression in roots, followed by stems and leaves, with lower expression in flowers, fruits and seeds. *PeHVA22B* was not detected in any tissues. *PeHVA22C* showed higher expression in fruits and roots, with lower expression in other tissues. *PeHVA22D* was the only gene with the highest expression level in stems, and relatively lower expressed in other tissues. Both *PeHVA22E* and *PeHVA22F* were predominantly expressed in fruits, followed by stems. Across all sampled tissues, *PeHVA22D* and *PeHVA22E* showed relatively abundant expression compared to other homologues (**Figure 6**). These findings suggested that *PeHVA22* genes may play roles in the development of different tissues in passion fruit.

### 3.8 Expression profile of *PeHVA22* genes during floral development of passion fruit

**Figure 7:**
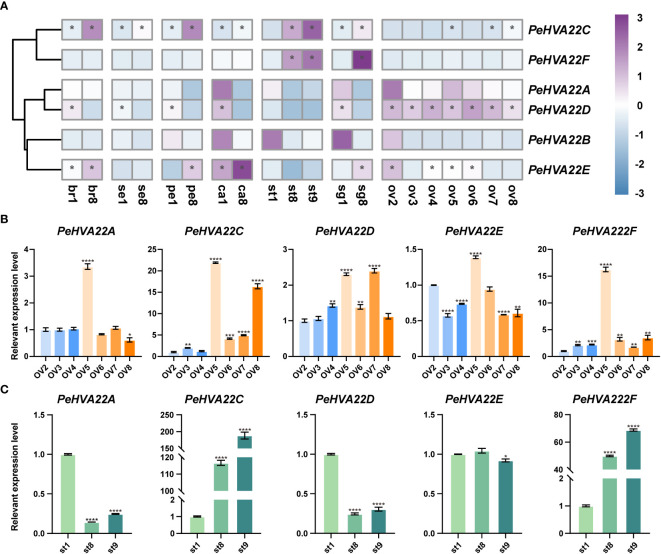
Expression profiles of *PeHVA22s* during floral development in passion fruit. **(A)** The heatmap was created based on the log_2_(TPM + 0.01) expression value of *PeHVA22s* and normalized by row. The TPM value higher than 50 was shown as abundant genes and marked with “*”. Differences in gene expression changes are shown in color as the scale, purple for high expression and blue for low expression. br, bract; se, sepal; pe, petal; ca, corona filament; st, stamen; sg, stigma; ov, ovule; numbers represent different developmental stages, 1 and 2 was early stage, 8 was late stage; **(B, C)** qRT-PCR analysis of *PeHVA22* genes in ovule and stamen development. Significant differences were analyzed by the Student’s t-test (*p-value < 0.05, **p-value < 0.01, ***p-value < 0.001, and **** p-value < 0.0001).

To understand the contributions of *PeHVA22* genes in reproductive development, RNA-seq data generated from early to mature stages of passion fruit floral samples, including bract (stages br1 and br8), sepal (stages se1 and se8), petal (stages pe1 and pe8), corona filament (stages ca1 and ca8), stamen (stages st1, st8 and st9), stigma (stages sg1 and sg8), ovule (stages ov2-0v8), was analyzed (**Figure 7A**, **Supplementary Table 5**). The results showed that *PeHVA22A* and *PeHVA22B* had low expression at the early stages of several tissues, such as petal, corona filament, stamen, stigma and ovule. *PeHVA22C* exhibited higher expression at the late developmental stages, with an ascending trend in all floral tissues including bract, sepal, petal, corona filament, stamen, stigma, and ovules. Conversely, *PeHVA22D* showed higher expression at the early developmental stages in almost all floral tissues except for stamen and ovule, where it was highly expressed throughout ovule development and nearly not expressed in stamens. *PeHVA22E* had high expression at the early developmental stages of the ovule and also higher expression at the late developmental stages of other tissues, including bract, petal and stigma. Besides, *PeHVA22F* was expressed with an ascending trend during the development of stamen and stigma.

The normal development of the ovule and stamen is a crucial prerequisite for the completion of sexual reproduction in passion fruit. Here, the expression patterns of *PeHVA22* genes during ovule and stamen development were further validated by qRT-PCR (**Figures 7B, C**). During ovule development, the relative expression trend of *PeHVA22* genes roughly aligned with the transcriptome data. For example, *PeHVA22A*, *PeHVA22D*, and *PeHVA22F* maintained relatively stable expression levels during ovule development. The expression level of *PeHVA22C* increased during ovule development. However, the relative expression level of *PeHVA22* genes in ovule development differed somewhat from transcriptome data. In the process of ovule development, most *PeHVA22* genes, expect for *PeHVA22D*, had their highest expression levels in ov5 stage. Similar to the transcriptome data, *PeHVA22C* and *PeHVA22F* were abundant at the late developmental stages of stamen with an ascending trend, while *PeHVA22A* and *PeHVA22D* were relatively repressed at the late developmental stages of stamen. There was no obvious change trend in the expression of *PeHVA22E* during stamen development. Overall, the qRT-PCR results were consistent with the transcriptome data analysis, indicating that some *PeHVA22* genes might play a role in stamen and ovule development in passion fruit.

## 4 Discussion

Passion fruit, an economically valuable fruit crop cultivated in tropical and subtropical regions globally, is highly susceptible to adverse climate conditions. The ABA signaling pathway plays a pivotal role in plant responses to abiotic stresses, regulating numerous stress-responsive genes to facilitate plants in coping with challenging environments. *HVA22* is a unique group of ABA-induced genes identified and reported to participate in the response to abiotic stresses in various plants, including Arabidopsis, citrus, tomato and cotton (Brands and Ho, 2002; Chen et al., 2002; Guo and Ho, 2008; Courtney et al., 2016; Gomes Ferreira et al., 2019; Wai et al., 2022; Zhang et al., 2023; Zhao et al., 2023). However, limited studies have explored the *HVA22* gene family in passion fruit.

In this study, six *PeHVA22* genes contained the TB2/DP1/HVA22 conserved domain were identified in the passion fruit genome. These genes were unevenly distributed across three pseudochromosomes, with four located on LG01 (**Figure 1A** and **Table 1**). The number of *PeHVA22* genes was relatively similar to that observed in citrus (six) (Gomes Ferreira et al., 2019), *Amborella trichopoda* (seven) and Arabidopsis (five) (Chen et al., 2002). However, it was fewer than the number in tomato (ranges from 14 to 16 in four different species) (Wai et al., 2022; Zhao et al., 2023) and cotton (ranges from 16 to 34 in different species) (Zhang et al., 2023). Previous studies have indicated that the expansion of the *HVA22* in tomato and cotton resulted mainly from gene duplication events. However, in this study, no such duplication events were identified in *PeHVA22* genes, similar to Arabidopsis (Chen et al., 2002). Therefore, the varying number of *HVA22* family members among different species might be attributed to the distinct evolutionary processes of these genes.

Previous studies have shown that *HVA22* homologs are widely found in diverse eukaryotes but absent in prokaryotes, indicating a unique role for *HVA22* in eukaryotes (Shen et al., 2001; Guo and Ho, 2008; Sharon and Suvarna, 2017; Fukuda et al., 2023). Subcellular localization analysis revealed that all PeHVA22 proteins were predicted to be situated within the endoplasmic reticulum (**Table 1**), consistent with the reported localization of many HVA22 proteins in plants, such as barley, Arabidopsis, and tomato (Guo and Ho, 2008; Chen et al., 2011; Lee et al., 2013; Wai et al., 2022). In rice, the overexpression of *OsHLP1* (HVA22-like protein 1) facilitated the disease resistance mechanism by perturbing endoplasmic reticulum homeostasis (Meng et al., 2022). Additional evidence in yeast also shown that the *HVA22* gene homolog *Yop1p* was involved in the transportation of substances between the endoplasmic reticulum and the Golgi apparatus (De Antoni et al., 2002). These results consistently implied that HVA22s played key roles as endoplasmic reticulum localization proteins in the cellular activities of diverse eukaryotes.

A maximum likelihood phylogenetic tree was constructed among passion fruit and five other species, and all HVA22 proteins could be mainly divided into four subgroups (**Figure 1B**). Consistent with previous work, Arabidopsis AtHVA22a, AtHVA22b and AtHVA22c were grouped in one subgroup while AtHVA22d and AtHVA22e were in the other subgroup with barley HVA22 (HvHVA22n), indicating the reliability of the classification results of our phylogenetic analysis. As to PeHVA22s, PeHVA22A and PeHVA22C were also clustered with barley HVA22 in Group II, PeHVA22B and PeHVA22D were clustered together in Group IV. Gene structural analysis was performed to further comprehend the development of the *PeHVA22* gene family. Among all *PeHVA22s*, the gene structural features of these two gene pairs (*PeHVA22A* and *PeHVA22C*, *PeHVA22B* and *PeHVA22D*) clustered together were similar and relatively conserved, while the gene structure characteristics of *PeHVA22s* from different subgroups varied significantly. The gene structure of *PeHVA22s* was correspondingly simple, with the number of exons ranging from 5 to 8 (**Figure 3A**), similar to the exon–intron amount in *AtHVA22*s and barley *HVA22* genes (Chen et al., 2002). However, we found that the size of the exons of *PeHVA22s* varied considerably (**Figure 3A**) and was inconsistent with the results in Arabidopsis, where all *AtHVA22* genes were reported to share a conserved exon size with barley *HVA22* genes (Chen et al., 2002). Additionally, the numbers and compositions of motifs also varied among different subclades (**Figure 3B**). Except for motif 2, which appeared in all PeHVA22 proteins, other motifs were tend to be specific to particular PeHVA22s, such as motif 4 being specific to PeHVA22A and PeHVA22C. The differences in gene structure characteristics could provide important sources for gene family diversification, resulting in different gene functions and expressions (Xu et al., 2012).

CREs scattered on gene promoters are closely related to the determination of gene regulation and functional roles (Hernandez-Garcia and Finer, 2014). Diverse CREs that respond to phytohormones (ABRE, GARE-motif, TATC-box, and P-box, etc.), environmental stresses (ARE, LTR, MBS, TC-rich repeats and WUN-motif, etc.) and plant growth/development (CAT-box, G-box, AE-box, TCT-motif, and GATA-motif, etc.) were identified within the promoter region of the *PeHVA22s* (**Figure 3C**, **Supplementary Tables 2**, **3**). The *HVA22* gene family, characterized as ABA-inducible, was found to have prevalent ABA responsiveness-related CREs in the promoter regions of almost all *PeHVA22s* except for *PeHVA22B*, and only abundant in *PeHVA22A* and *PeHVA22C*. Combining with expression analysis results, the expression of *PeHVA22B* was not detectable in all the test samples by qRT-PCR (**Figure 5**). Moreover, the transcriptome data showed that the expression of *PeHVA22B* was also at a relatively low level during diverse floral organ development. Thus, we speculated that *PeHVA22B* may be a pseudogene, which was required for further verification. Following ABA treatment, the expression of *PeHVA22A* and *PeHVA22C* was significantly enhanced at 24 h after treatment while other genes were suppressed after treatment. Different changes in the expression of *HVA22* genes in response to ABA have also been reported in Arabidopsis (Chen et al., 2002). The presence of multiple identical ABA response elements in the promoter regions might contribute to more intense responses of *PeHVA22A* and *PeHVA22C* to ABA signaling. In addition, GA-responsive related elements were also common in the promoter region of the *PeHVA22* genes, and we found that the expression of most *PeHVA22* genes was enhanced by GA except for that of *PeHVA22B* (not detectable) and *PeHVA22F* (suppressed). Barley *HVA22* was reported to be involved in the negative regulation of GA-mediated programmed cell death and vacuolation in aleurone layer cells (Guo and Ho, 2008). These results uncovered that the regulation of *HVA22* gene expression by GA signaling may be also common and important. Furthermore, the expression profiles of *PeHVA22s* under stress treatments were also explored (**Figure 5**). In general, the expression of most *PeHVA22s* was induced at a certain time after drought treatment, while suppressed under other treatments including salt, low and high temperature, implying that *PeHVA22* genes might play a unique role in drought response. TFs have important roles in plant stress response and growth and development. Here, the potential TFs targeting the *PeHVA22* genes were predicted, and the regulatory network interactions with the *PeHVA22* genes were constructed (**Figure 4** and **Supplementary Table 4**). Many TFs involved in stress response were identified such as *NAC, Dof* and *bZIP* TFs. In rice, *OsHLP1* (HVA22-like protein 1) was reported to interact with the *NAC* transcription factor *OsNTL6* to initiate blast disease resistance by modulating endoplasmic reticulum homeostasis (Meng et al., 2022). Consistent with previous investigations, these findings indicate that the role of TFs in *PeHVA22* genes may be important for the regulation of plant stress tolerance.

In plants, *HVA22* is expressed in various tissues, for example, roots, stems, leaves, and seeds (Shen et al., 1993; Shen et al., 2001; Chen et al., 2002; Gomes Ferreira et al., 2019; Wai et al., 2022). Distinct expression patterns of *PeHVA22* genes across various tissues indicate potential variations in their regulatory roles during the growth and development of passion fruit (**Figure 6**). *PeHVA22A* and *PeHVA22D* had high expression levels in vegetative tissues (root and stem), whereas the relative expression level of *PeHVA22C*, *PeHVA22E*, and *PeHVA22F* were higher in reproductive organs (fruit and flower), implying these genes’ probable roles in growth and developmental processes in passion fruit. *PeHVA22C*, *PeHVA22E*, and *PeHVA22F* were dominantly expressed in fruits and their homolog *SlHVA22C/I/K/L/N* had the highest expression levels in tomato fruit, suggesting their preferential role in fruit development (**Figure 1B**, **Figure 6**) (Wai et al., 2022). In various species, *HVA22* genes are also reported to play pivotal roles in developmental processes associated with plant flower, female and male (Chen et al., 2002; Chen et al., 2009; Gomes Ferreira et al., 2019; Li et al., 2021). Passion fruit, being a popular ornamental plant, offers a unique flower structure suitable for investigating flower development. To explore the potential contribution of *PeHVA22* genes in flower organ development, we examined the expression levels of all *PeHVA22s* across different tissues of passion fruit flower organs. The results revealed distinct expression profiles among *PeHVA22* genes, with variations in expression levels observed for the same gene across different floral tissues (**Figure 7A**, **Supplementary Table 5**). Some *PeHVA22s* were concurrently highly expressed in multiple flower tissues, indicating redundant functions in flower organs. For example, *PeHVA22C* had high expression in the later stages of bract, petal, and stamen, while *PeHVA22D* showed elevated expression in the early stages of bract, sepal, petal, corona filament, and stigma, and expressed throughout ovule development. Moreover, all *PeHVA22* genes were expressed in different developmental stages of the stamen or ovule, which was also verified by qRT-PCR results (**Figures 7B, C**). These findings align with observations in *HVA22* genes of other species (Schneitz et al., 2002; Li et al., 2021), indicating that *HVA22* may play a crucial role in the development of stamen and ovule in passion fruit. Additionally, we found that almost all *PeHVA22* genes were both stress-responsive and also preferentially expressed in certain tissues at specific developmental stages. Various TFs related to phytohormone signaling and plant growth/development, including *LBD*, *MIKC_MADS*, *SBP* and *ERF*, were also found in the regulation of *PeHVA22* genes. Taken together, the complex regulation of TFs and CREs might contribute to the multiple roles of *PeHVA22* genes in different processes.

## 5 Conclusions

In this study, we identified six *HVA22* genes in passion fruit, distributed unevenly across three chromosomes. Through analyses encompassing phylogenetic trees, gene structures, and conserved motif examination, we found that closely related gene members tend to show similar exon/intron structures and motif compositions. Analyses of CREs prediction and qRT-PCR results under various treatment conditions suggested that *PeHVA22s* might responded to different plant hormones and stresses. Expression analysis in different tissues revealed tissue-specific expression patterns for *PeHVA22* genes. For instance, *PeHVA22A* was specifically expressed in root, while *PeHVA22C/E/F* had the highest expression in fruit, suggesting their specific roles in the development of certain organs. Based on the expression profiles of *PeHVA22s* in floral tissues, these *PeHVA22* genes exhibited significant differences among various flower tissues, with distinct expression levels for the same gene in different floral tissues. Collectively, our comprehensive analyses provide valuable insights into the evolutionary trajectory of the *PeHVA22* gene family and significantly enhance ongoing research into the functional attributes of *PeHVA22* in flower tissue development and responses to diverse stresses.

## Data availability statement

The datasets presented in this study can be found in online repositories. The names of the repository/repositories and accession number(s) can be found in the article/**Supplementary Material**.

## Author contributions

PZ, YQ and ZH: Designed experiments, revised manuscripts, and obtained funds. JXL, XC, RL and SC: Performed the bioinformatics analysis and review & editing manuscripts. JTL: Drawn the concept figure of passion fruit and review & editing manuscripts. ZH, AC, XW and GC: Collected the different tissues samples, different stages of ovule and stamen, performed the qRT–PCR and review & editing drafts. ZH and LL: Performed the experiments of stress treatments and review & editing manuscripts. ZH, JXL and PZ: Writing the original manuscript. PZ and YQ: reviewed and edited the manuscript. All authors contributed to the article and approved the submitted version.
